# Centro-Apical Self-Organization of Organic Semiconductors in a Line-Printed Organic Semiconductor: Polymer Blend for One-Step Printing Fabrication of Organic Field-Effect Transistors

**DOI:** 10.1038/srep14010

**Published:** 2015-09-11

**Authors:** Su Jin Lee, Yong-Jae Kim, So Young Yeo, Eunji Lee, Ho Sun Lim, Min Kim, Yong-Won Song, Jinhan Cho, Jung Ah Lim

**Affiliations:** 1 Center for Opto-Electronic Materials and Devices, Post-Silicon Semiconductor Institute, Korea Institute of Science and Technology (KIST), Seoul, 136-791, Korea; 2 Department of Chemical and Biological Engineering, Korea University, Seoul, 136-713, Korea; 3 Graduate School of Analytical Science and Technology, Chungnam National University, Daejeon, 305-764, Korea; 4 Electronic Materials and Device Research Center, Korea Electronics Technology Institute, Gyeonggi-do, 463-816, Korea; 5 Department of Chemical Engineering, Pohang University of Science and Technology (POSTECH), Pohang, 790-784, Korea

## Abstract

Here we report the first demonstration for centro-apical self-organization of organic semiconductors in a line-printed organic semiconductor: polymer blend. Key feature of this work is that organic semiconductor molecules were vertically segregated on top of the polymer phase and simultaneously crystallized at the center of the printed line pattern after solvent evaporation without an additive process. The thickness and width of the centro-apically segregated organic semiconductor crystalline stripe in the printed blend pattern were controlled by varying the relative content of the organic semiconductors, printing speed, and solution concentrations. The centro-apical self-organization of organic semiconductor molecules in a printed polymer blend may be attributed to the combination of an energetically favorable vertical phase-separation and hydrodynamic fluids inside the droplet during solvent evaporation. Finally, a centro-apically phase-separated bilayer structure of organic semiconductor: polymer blend was successfully demonstrated as a facile method to form the semiconductor and dielectric layer for OFETs in one- step.

As recent advances in organic field-effect transistors (OFETs) based on solution-processed organic semiconductors have achieved high performances with field-effect charge mobilities over 20 cm^2^V^−1^s^−1^, research efforts have been focused on the development of a novel printing technique for organic semiconductors to incorporate their devices into large-area electronics^1^,^2^. One of the most fascinating aspects of organic semiconductors is that unlike other semiconductor materials, the semiconductor channel can be easily formed at room temperature by direct-write printing such as gravure printing and inkjet printing^3^,^4^,^5^. However, in practice, organic semiconductors are fastidious materials to handle with respect to printable semiconductor ink because the charge carrier transport in the organic semiconductor is dictated by the morphology and molecular orientation of their organic semiconductor films. Furthermore, the printed feature is strongly dependent on the printing conditions, such as the solvents, solution concentration, and surface wettability of the substrate^6^,^7^,^8^,^9^,^10^. Therefore, the printing of organic semiconductors with the desired molecular orientation and uniform morphology has remained an important challenge to perfect this technology.

Recently, organic semiconductors blended with polymeric binders have been the topic of intensive investigations as a promising approach to formulate organic semiconductor ink^11^,^12^,^13^,^14^,^15^. In particular, blending polymeric binder is an important strategy for formation of uniform films with small-molecule organic semiconductors, such as 6,13-bis(triisopropylsilylethynyl) pentacene (TIPS-PEN), triethylsilylethynyl anthradithiophene (TES-ADT), 2,8-difluoro-5,11-bis(triethylsilylethynyl)anthradithiophene (diF-TESADT), and 2,7-dioctyl[1]benzothieno[3,2-b][1]benzothiophene (C8-BTBT)^4^,^16^,^17^,^18^,^19^,^20^. Although such small-molecule organic semiconductors exhibit intrinsically high charge carrier mobilities and good solubility in organic solvents, film dewetting cuased by strong pi-pi interactions and large device-to-device performance variations have been pointed out as critical drawbacks. Blends of polymer and small-molecule organic semiconductors have been successfully utilized in combination to create excellent film-forming properties inherent with polymers with the excellent charge carrier mobilities found in the organic semiconductor crystalline structure. However, in such blend films, the vertical segregation of the organic semiconductor molecules toward the air/film interface (or substrate/film interface) has been still critical issue in guaranteeing an electronic percolation pathway because the charge carriers are efficient with transport in the organic semiconductor phase and their transport occurs in parallel to the substrate in the OFET device^20^. Since the phase-separation behavior is governed by many factors, such as material compatibility, the solvent evaporation rate, and the surface wettability of the substrate, the process conditions should be fine-tuned to obtain vertical phase-separation in the polymer blends. Nevertheless, the vertically segregated bilayer structures is still fascinating considering that separate functional layers of the semiconductor and the gate-dielectric or semiconductor and the top-passivation layer in the OFET device can be deposited in a one-step film deposition process^21^,^22^. To date, although the majority of studies report a vertically segregated bilayer structure that is mainly comprised of spin-coated thin films, the identical phase-separation behavior in printed blend patterns has not been demonstrated. Recently, Cho *et al.* reported on the vertical phase-separation behavior in an inkjet-printed TIPS-PEN/polymer blend, where the movement of the TIPS-PEN molecules toward the air/film interface was attributed to the improvement in the hole mobilities of the OFETs^11^. However, in their work the polymers functioned only as a binder where the underlying polymer matrix did not serve as a gate dielectric in OFET device.

In this work, we present a one-step printing method of organic semiconductor channels and polymer dielectric bilayers for an OFET device by controlling the self-organization of the organic semiconductor/dielectric polymer blend. Key feature of our finding is that the organic semiconductor molecules were vertically segregated onto the top of polymer phase and simultaneously crystallized at the center region of the printed line pattern just after the solvent evaporation process without an additive process. We justify this self-organization of the organic semiconductor in the confined blend solution through the use of the term “centro-apical self-organization” throughout the remainder of the paper. To the best of our knowledge, this is the first report on the vertically segregated and center-aligned organic semiconductors in a printed organic semiconductor/polymer blend pattern and their application to OFET devices. Thicknesses and widths of the centro-apically segregated organic semiconductor crystalline structure in the printed blend pattern were controlled by varying the relative content of the organic semiconductors in the blend solution. The influences of the printing speeds and blend solution concentrations on the centro-apical phase separation were systematically investigated. The actual OFETs consisted of the centro-apically phase-separated organic semiconductor channel and a gate dielectric of an underlying polymer matrix exhibited a good performance approaching previously reported values of devices prepared via either the spin-casting or drop-casting methods.

## Results

### Characterization of the Centro-Apical Self-Organization in the Printed diF-TESADT:PMMA Blend

**Figure 1:**
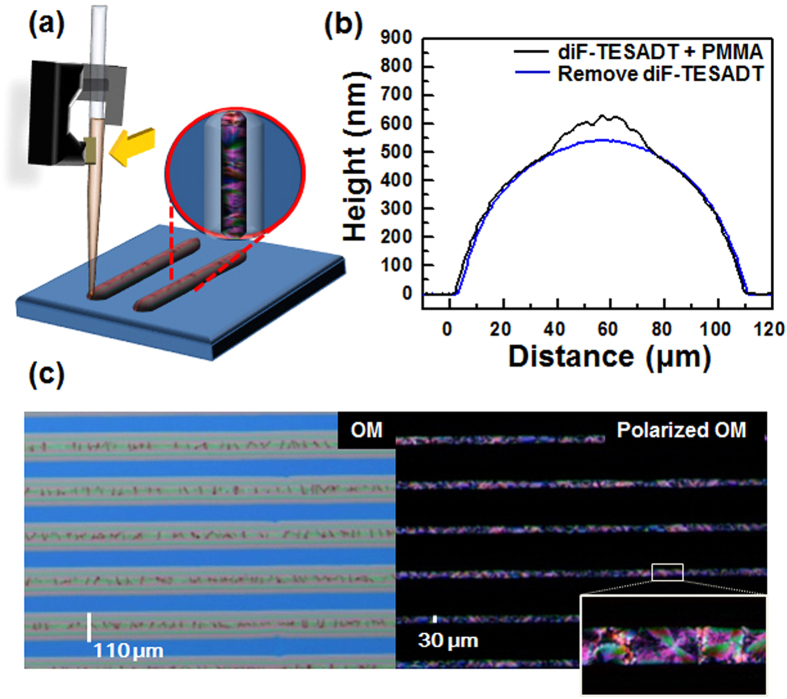
(**a**) Schematic of the printing process using the picoliter fluidic dispenser with a magnification of the printed line patterns. (**b**) Comparison of the height profiles of the printed blend line before and after the selective removal of diF-TESADT by cyclohexane. (**c**) OM and POM images of the diF-TESADT:PMMA (10 wt% and a 1:4 w/w ratio) printed lines with a printing speed of 400 μm/s. The width of the entire printed line is approximately 110 μm, and the width of the diF-TESADT crystalline stripe at the center region is approximately 30 μm.

2,8-Difluoro-5,11-bis(triethylsilylethynyl)anthradithiophene(diF-TESADT), a small molecule semiconductor with a large charge carrier mobility, was blended with an insulating polymer, poly(methyl methacrylate) (PMMA). 10 wt% solution of the 1:4 blend of diF-TESADT and PMMA was printed onto a UV-ozone-treated silicon wafer using a picoliter fluidic dispenser, where the ultrasonic vibrations cause the fluid to be ejected from the tip of the micropipette and to form a fluidic meniscus at the end of the tip that is touching the surface of the substrate (micropipette tip is placed 5 ~ 10 μm above the substrate) (Fig. 1a)^23^. Unlike with inkjet printing which struggles with dispensing highly viscous solutions (i.e., high-concentration solutions), the picoliter fluidic dispenser allows for the deposition of micro-patterns with high-aspect-ratios from the one-step printing of a highly concentrated solution. The viscosity of t he blend solution used in this work was approximately 1,000 cP, which is virtually unusable in inkjet printing. 1,2,4-trichlorobenzene (TCB), with its high boiling point and low vapor pressure, was chosen as a solvent. The higher boiling point of the solvent ensures stable printing without clogging of the tip end. It also, provides more time for the phase separation or self-organization of the molecules in the printed solution during the solvent evaporation^13^.

Figure 1c presents representative optical microscope (OM) images of the printed diF-TESADT:PMMA (1:4) blend pattern. The width of the entire printed line is approximately 110 μm. Interestingly, spherulitic-like crystals, particularly positioned at the center region of the printed line, were observed from a polarized OM image (POM). The crystalline domains were uniformly formed along the length of the printed line and appeared as a separate crystalline stripe inside the printed line. The central crystalline structure clearly originated from the crystallization of the diF-TESADT molecules because atactic PMMA has an amorphous nature. The diF-TESADT crystalline stripe is approximately 30 μm wide at the center region. As shown in Fig. 1b, the printed blend pattern has a bell-shaped height profile, where the center region consisted of diF-TESADT crystals that rose upward. In contrast, after a selective dissolution of diF-TESADT by dipping in cyclohexane, the height profile of diF-TESADT exhibits a smooth, hemispherical shape. The thicknesses of the top-segregated diF-TESADT and underlying PMMA-dominant layer were approximately 50 and 550 nm, respectively. This result indicates that the diF-TESADT molecules were dominantly segregated toward the upper film/air interface and were simultaneously crystallized along the centerline of the cylindrical pattern of the printed blend solution.

To confirm the presence of vertically separated and self-centered diF-TESADT molecules in the printed polymer blend, a cross-sectional SEM analysis was performed on the printed line with the 1:4 ratio diF-TESADT:PMMA blend (Fig. S1 in the supplementary information (SI)). The SEM image clearly shows the distinct layer boundary, which is derived from the vertical phase separation. EDS analysis focusing on each layer indicated that the top layer possessed a higher percentage weight of sulfur that originated from diF-TESADT than that taken from the bottom layer. (A detailed explanations for SEM analysis is provided in SI.) In addition, time of flight secondary ion mass spectroscopy (TOF-SIMS) combined with local cesium ion beam sputtering was performed to investigate the distribution of the diF-TESADT molecules at the vertically segregated center region (Fig. S2 in SI). We confirmed that the top layer of the central crystalline regime predominantly consisted of diF-TESADT molecules and a PMMA-rich region with diF-TESADT as a minor component was observed near the substrate (A detailed explanations for TOF-SIMS analysis is provided in SI.). The somewhat remanent distribution of diF-TESADT within PMMA phase could be attributed to the high viscosity of the blend solution used in this work, which may provide an environment in which the diF-TESADT molecules cannot easily move toward the air/film surface. In previous literature, Cho *et al.* also demonstrated that vertical phase-segregation with the clear layer boundary occurred when a low-viscosity TIPS-pentacene/polymer blend solution was used for inkjet printing^11^. Although the broad coexistence layer was formed, the underlying region could successfully function as a bottom gate dielectric layer because the diF-TESADT concentration was below the percolation threshold. In fact, the leakage current density measured from a capacitor based on the printed blend pattern after the removal of the top diF-TESADT molecules using cyclohexane indicated that the PMMA-rich underlying layer did not undergo dielectric breakdown until 100 V, which corresponds to a breakdown field of 1.8 MV cm^−1^ (Fig. S3a in SI).

**Figure 2:**
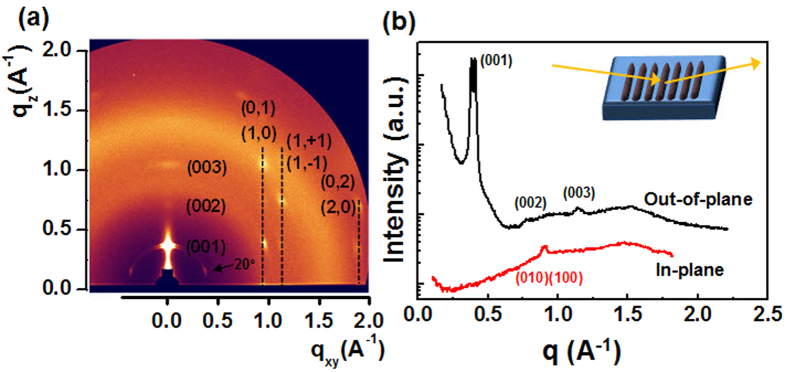
(**a**) 2-dimensional grazing incidence X-ray diffraction (2D GIXD) patterns of the diF-TESADT: PMMA-blend printed lines. (**b**) Out-of-plane and in-plane X-ray diffraction patterns of the diF-TESADT: PMMA-blend printed lines. The incident beam was injected perpendicular to the printed line patterns.

In order to observe the molecular orientation of the diF-TESADT:PMMA blend printed line two-dimensional grazing incidence X-ray diffraction (XRD) was performed. The incident beam was injected perpendicular to the printed line pattern, as shown in the inset of Fig. 2b. Figure 2a illustrates that the diF-TESADT:PMMA printed line contained (001) diffraction peaks at *q*_*xy*_ = 0, as well as many reflection spots along *q*_*z*_ for a given *q*_*xy*_. The out-of-plane (001) diffraction peaks and strong in-plane (010) and (100) diffractions indicate that the diF-TESADT molecules in the printed blend line stacked with the silyl groups on the substrate^24^. This edge-on molecular orientation implies that the diF-TESADT molecules have a π-π stacking structure parallel to the substrate, which is the desired orientation for efficient charge transport in OFET devices. The observed two split peaks at the (001) diffraction, with one at a distance of d = 16.3 Å and the other one at d = 15.3 Å, may be due to the polymorphic structure of the triclinic diF-TESADT crystal. It was reported that vertically segregated diF-TESADT molecules from the PMMA blend pack in layered stacks oriented to the surface normal with a d-spacing of 15.8 Å, which is smaller than the d-spacing of bulk phase (16.3 Å)^15^. The weak (001) features at nominally 20° from the horizon originated from the tilted orientation with respect to the surface normal. The occurrence of the 20° tilted structure might be related to the tilting of the fluorinated anthradithiophene backbone in the solvent evaporation of the printed blend solution. The influence of the solvent evaporation rate on the phase separation and crystalline structure of diF-TESADT in the blend film has been reported previously; the lying-down or 20° tilted structures were kinetically stable and were observed when the films were prepared under rapid solvent evaporation^13^. Additionally, an identical XRD pattern was observed when the incident beam was injected parallel to the direction of printed blend line. (Fig. S4 in SI) This confirms that the molecular orientation of diF-TESADT is not dependent on the printing direction.

### Controlling the Geometries of the Centro-Apically Phase-Separated diF-TESADT:PMMA Bilayer structure

**Figure 3:**
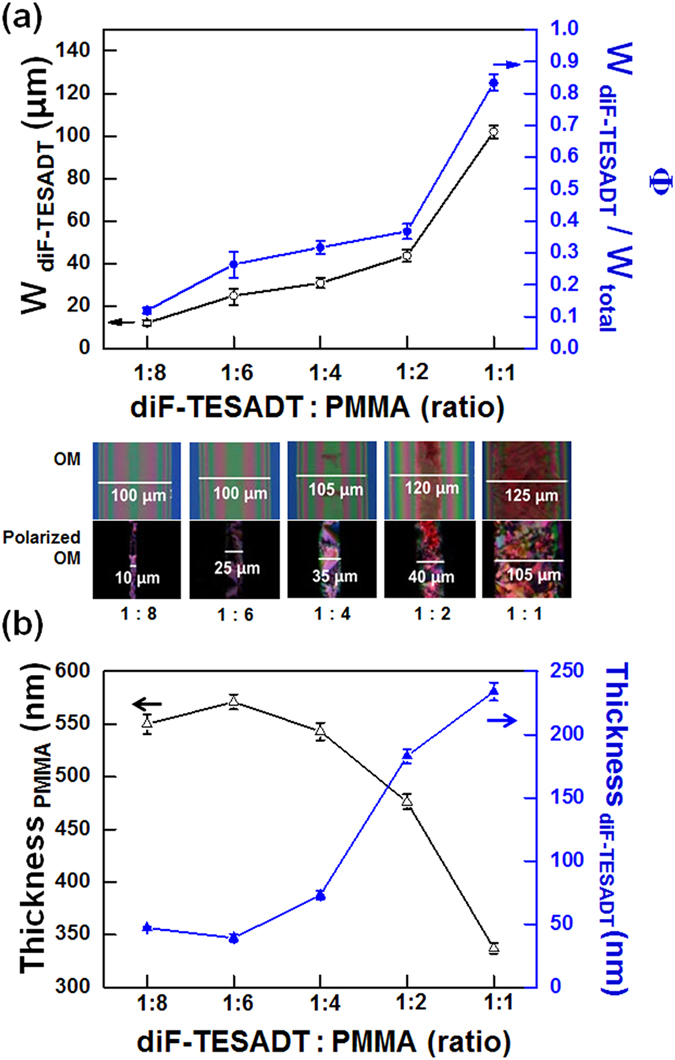
(**a**) Relationships between varying the diF-TESADT:PMMA ratios (bottom) and line -widths of the centro-apically segregated diF-TESADT crystalline (W_diF-TESADT_) (left) and the ratios (Φ) of W_diF-TESADT_ to W_total_ (right). Corresponding OM (top) and POM (bottom) images are displayed below the plot. (**b**) Thicknesses of the centro-apical segregated diF-TESADT (right) and underlying dominant PMMA layer (left) with respect to the variation in the different blend ratios.

We investigated the possibility of controlling the geometries of each component in the phase-separated films by varying the diF-TESADT:PMMA blend ratio. At a fixed concentration of 10 wt%, batches of the solution with different diF-TESADT:PMMA ratios were prepared and printed at the same printing speed of 400 μm/s. Figure 3a shows the line-width of the centro-apically segregated diF-TESADT crystalline (W_diF-TESADT_) plotted on various diF-TESADT:PMMA ratios with corresponding OM images displayed below the plot. The total line-widths of the blend patterns (W_total_) gradually decreased with a corresponding increase in the PMMA concentration in the blend solution. This is attributed to an increase in the solution viscosity; highly viscous solutions spread slowly on the substrate after deposition, which can result in printed line-widths that are thicker and narrower than those obtained from a less viscous solution. Interestingly, the line-width of the center-aligned diF-TESADT (W_diF-TESADT_) increased as the amount of diF-TESADT in the blend solution increased. The ratio (Φ) of W_diF-TESADT_ to W_total_ clearly shows that the increase in W_diF-TESADT_ is predominantly determined by the diF-TESADT:PMMA blend ratio, but it is not related to the increase in W_total_. At the 1:1 diF-TESADT:PMMA blend ratio, nearly 85% of W_total_ was covered by the diF-TESADT crystals. In contrast, at the 1:8 diF-TESADT:PMMA blend ratio, the center-aligned diF-TESADT occupied only 12% of W_total_, which corresponds to approximately 12 μm (±2 μm). This result indicates that the resolution of the printed organic semiconductor channel can be controlled by changing the blend ratio. To date, although the printing of organic semiconductors has been intensively demonstrated using various methods to fabricate organic transistors, printed features were unnecessarily overwhelmed in the actual channel region of the thin-film transistors (TFTs). To the best of our knowledge, this is the first demonstration of directly-printed well-defined organic semiconductor channels of ~10 μm, which is below the limit resolution of the general printing methods (~20 μm). Additionally, the thicknesses of the centro-apical segregated diF-TESADT and underlying PMMA-dominant layer varied with different blend ratios, as shown in Fig. 3b. As the diF-TESADT content increased, the thickness of the diF-TESADT layer increased from 50 nm to 250 nm, whereas the thickness of the underlying dominant PMMA layer decreased from 550 nm to 330 nm. This result again supports the fact that the diF-TESADT molecules tend to centro-apically transport in a printed blend pattern during the solvent evaporation process.

**Figure 4:**
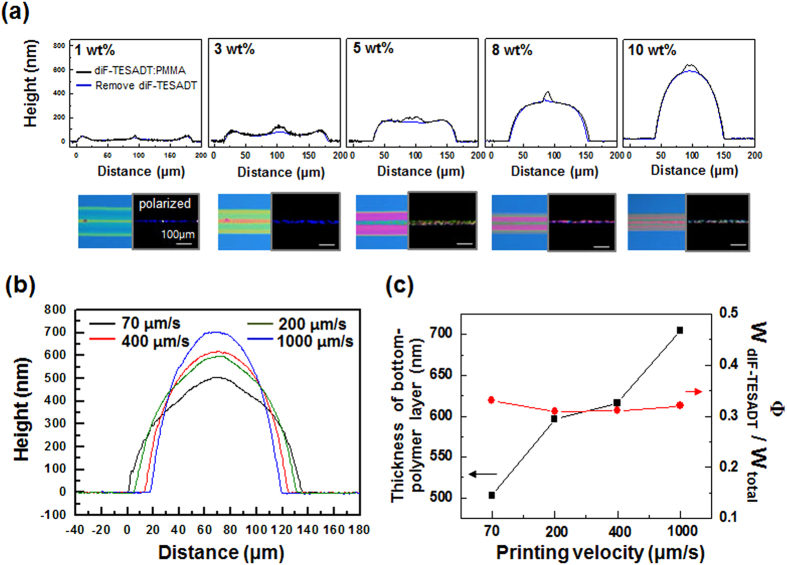
(**a**) Comparison of the height profiles of the printed line (black) before and after the selective removal of the diF-TESADT (blue) with a variation in the concentration of the diF-TESADT:PMMA (1:6 wt/wt ratio) solution. Below the graph, the images show the OM and POM. (**b**) Profiles of the PMMA phase regarding the variation in the picoliter fluidic dispenser printing velocity. (**b**) Thickness of the bottom-PMMA layer and the ratio (Φ) of W_diF-TESADT_ to W_total_ with respect to the variation in the printing velocity.

To check whether the central gathering of crystal in the printed line during the drying process only occurred in highly viscous solutions, 1:6 diF-TESADT:PMMA blend solutions at concentrations of 1, 3, 5, 8, and 10 wt% were printed. As shown in Fig. 4a, all of the height profiles of the printed blend line obtained from different concentrations before and after the selective dissolution of diF-TESADT showed formations of the centerline-placed diF-TESADT crystalline. W_diF-TESADT_ was not significantly changed by varying the total concentrations of the blend solutions. 1:4 diF-TESADT:PMMA blend solutions also showed similar tendency with a variation in the total concentration of blend solutions. (Fig. S5 in SI) This indicates that the content of diF-TESADT in the blend solution is the predominant factor in determining the line-width of the center-aligned crystalline stripe. In contrast, a higher concentration of blend solution results in a thicker bottom-polymer layer with a smaller line width. This means that the thickness of the bottom gate dielectric layer for transistor applications can be independently controlled by varying the solution concentration without changing the feature size of the diF-TESADT centerline. Figure 4b,c show influence of the printing velocity on the centro-apical phase-separation of the blend. As the printing velocity increased from 70 to 1,000 μm/s, the thickness of the bottom-polymer layer increased and the total line width decreased, whereas Φ remained nearly constant. These results indicate that the dimension of each layer in cento-apically separated bilayer could be selectively controlled by varying the relative contents of the organic semiconductor and polymer, the solution concentration, and the printing speeds.

### Electrical Performance of the One-Step Printed OFETs Based on the Centro-Apical Phase-Separation of the diF-TESADT:PMMA Blend.

**Figure 5:**
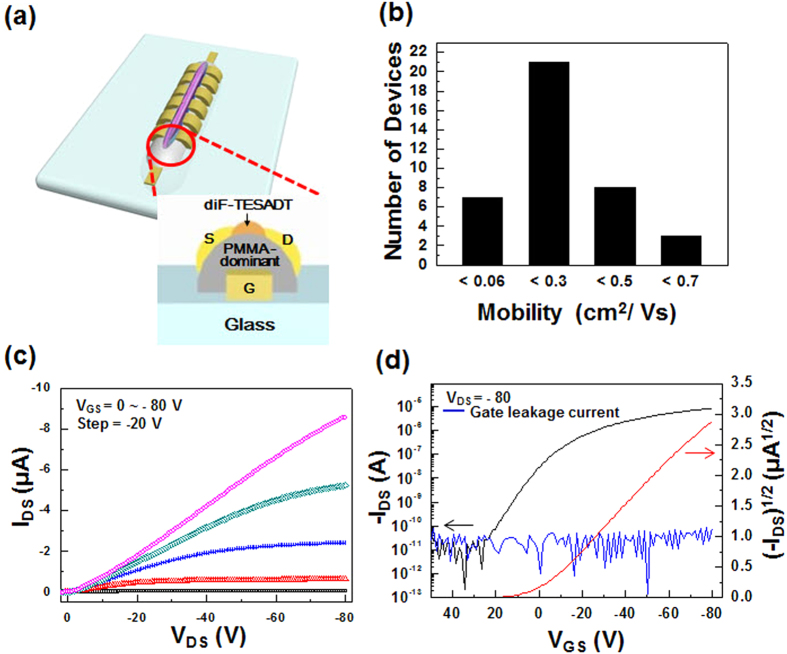
(**a**) Schematic diagram of the front and side views of the device structure. (**b**) Statistical distribution of the field-effect mobilities. (**c**) Output (I_DS_–V_DS_) and (**d**) transfer (I_DS_–V_GS_) characteristics (at V_DS_ = −80 V) of a diF-TESADT:PMMA (1:4 w/w ratio) blend printed line transistor.

Top-contact and bottom gate OFETs were fabricated to evaluate the electrical characteristics of the OFETs based on the one-step printed semiconductor/polymer dielectric bilayer formed by the phase-separation of diF-TESADT:PMMA. The images in Fig. 5a are the front and side views of the device structure. Figure 5c,d shows the output and transfer characteristics of the one-step printed OFETs. Although the diF-TESADT molecules coexisted in the PMMA -bottom layer, the gate leakage currents were extremely low, i.e., in the range of 10^−12^ ~ 10^−9^ A, for all devices, as shown in the transfer curve. The capacitance of the underlying PMMA-dominant layer, measured after the selective dissolution of diF-TESADT, was approximately 3.5 nF/cm^2^ (Fig. S3b in SI). The average field-effect mobility and on/off current ratio calculated from the transfer characteristics in the saturation regime were 0.33 cm^2^V^**−**1^s^**−**1^ and ~10^5^, respectively. This performance closely corresponds with the previously reported values of devices based on spin-casted diF-TESADT:PMMA blends^13^,^15^,^21^. Two types of devices with different source and drain electrode arrangements (i.e. parallel and perpendicular to the printed line) exhibited no significant differences in the electrical performances (Fig. S6 in SI), as expected based on the fact that centro-apically separated diF-TESADT formed the spherulitic crystalline with isotropic molecular orientation. To examine the reproducibility of the device, the electrical properties of 40 devices were tested. The device yield was over 90%. Figure 5b presents the statistical distribution of the field-effect mobilities, and the highest performance achieved by this method was 1.06 cm^2^V^**−**1^s^**−**1^ with an on/off current ratio of ~10^4^. (Fig. S7 in SI) These results clearly show that one-step printing of diF-TESADT:PMMA successfully provides an organic semiconductor channel as well as a bottom gate dielectric layer without an additive process.

**Figure 6:**
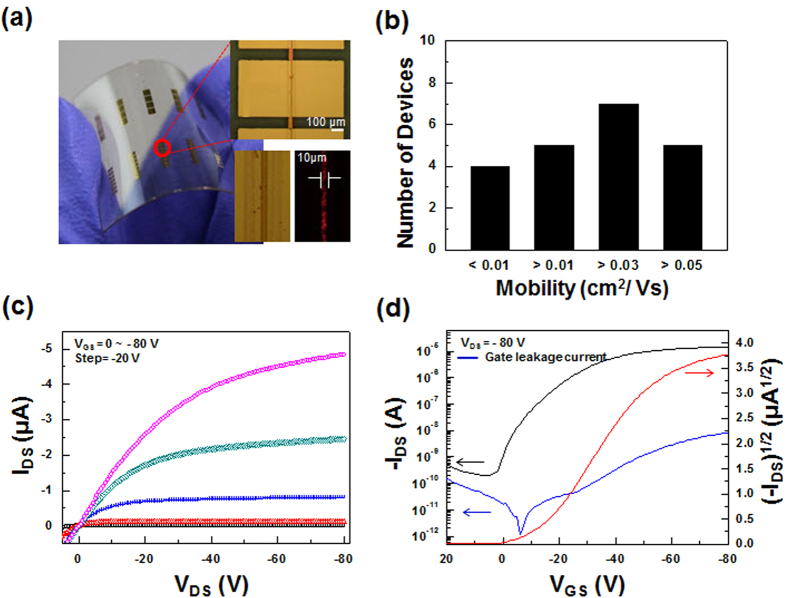
(**a**) Photo image (left) and OM images (right) of a diF-TESADT:PMMA (1:8 w/w ratio) blend printed line transistor array on a PES substrate. (**b**) Statistical distribution of the field-effect mobilities. (**c**) Output (*I*_DS_–*V*_DS_) and (**d**) transfer (*I*_DS_–*V*_GS_) characteristics (at *V*_DS_ = −80 V) of a diF-TESADT:PMMA (1:8 w/w ratio) blend printed line transistor.

Additionally, we verified the feasibility of this method to fabricate flexible devices and direct-print organic semiconductor channels that were well defined within channel-lengths of as low as 10 μm. Flexible OFETs with short channel lengths were constructed on a polyethersulfone (PES) substrate by printing a 1:8 diF-TESADT:PMMA blend solution, as shown in Fig. 6a. As shown in Fig. 6c,d, the output and transfer characteristics of the devices exhibited a gate-bias response, and there was no contact barrier for charge injection from the electrodes. This verified that the centro-apically segregated diF-TESADT molecules from a 1:8 diF-TESADT:PMMA blend solution were densely packed and formed a crystalline film for the efficient transport of charge carriers. The average field-effect mobilities and on/off current ratios of these flexible OFETs with a 10 μm channel length were approximately 0.03 cm^2^V^**−**1^s^**−**1^ and 10^5^, respectively. When the devices were fabricated on the glass substrate using a 1:8 diF-TESADT:PMMA blend solution the device performances were consistent with that of the devices based on the flexible substrate. (Fig. S8 in SI) The best performance of the one-step printed flexible OFET has field-effect mobility of 0.07 cm^2^V^**−**1^s^**−**1^ and on/off current ratio of 10^7^. Relatively lower mobility of this device compared with the value of the devices prepared from the 1:4 diF-TESADT:PMMA blend solution might be due to an incomplete phase-separation and less-ordering of the diF-TESADT blend solution with a small amount of diF-TESADT in the viscous blend solution because the self-organizing movement of the diF-TESADT molecules in the more viscous solution could be restricted^11^. The transistor performance of the device printed from the 1:8 diF-TESADT:PMMA solution will be improved by adjusting the viscosity of the blend solution.

## Discussions

To obtain further insight into the origin of centro-apical phase-separation, we observed the evolution of the printed blend solution features during solvent evaporation using a CCD camera equipped with the dispenser cartridge. The live video of the drying of the printed line is available in the supplementary information. Approximately 60s later, the nucleation of the diF-TESADT crystals was inceptively observed at the starting point of the printing, and the central crystallization of diF-TESADT occurred continuously along the line-printing direction. This implies that during solvent evaporation, the diF-TESADT molecules moved toward the apex position of the printed droplet and then self-assembled at the center region. This centro-apical movement of the diF-TESADT molecules in a blend droplet is likely due to the combination of the vertical phase-separation, which is an energetically favorable transition, and the influence of the hydrodynamic fluids inside of a cylindrical droplet during solvent evaporation.

**Figure 7:**
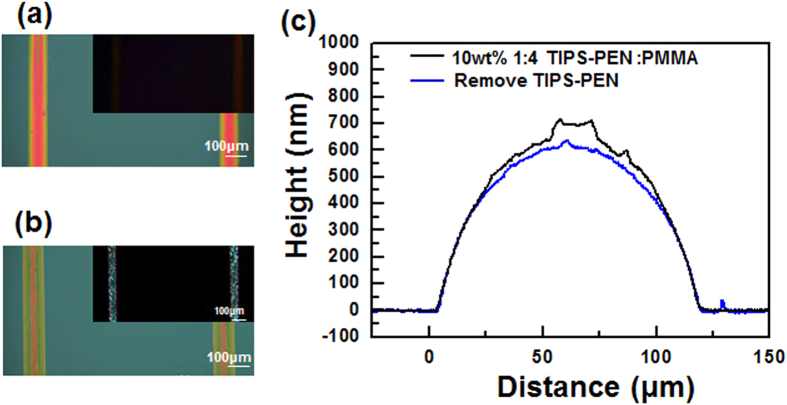
(**a**) OM image (inset : POM image) of the diF-TESADT:PS printed lines. (**b**) OM image (inset : POM image) of the TIPS-PEN:PMMA printed lines. (**c**) Comparison of the height profiles of the printed line before (black) and after (blue) the selective removal of TIPS-PEN by hexane.

The compatibility of diF-TESADT and PMMA is one of the most important factors for the vertical phase-separation in the blend. As is well known, the miscibility of a blend can be estimated by using the Flory-Huggins interaction parameter (χ)^25^,^26^. The value of χ has the following relationship with the difference between the solubility parameters (*δ*) of each component, 
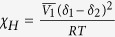
. As the difference of the solubility parameters 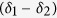 increases, the χ becomes a larger positive value, making phase separation more likely. A solubility parameter of approximately 15.5 MPa^1/2^ was calculated the diF-TESADT using group contribution method (see supplementary information Table S1)^27^. Table 1 summarizes the solubility parameters of small-molecule semiconductors of diF-TESADT and TIPS-PEN, as well as those of the insulating polymers of polystyrene and PMMA. The large difference between the solubility parameter of the semiconductor materials and that of PMMA (22.69 MPa^1/2^) supports the theory that the phase-separation is energetically favorable in this blend. As a comparison, when polystyrene (PS) (*δ* = 17.52 MPa^1/2^), which has a solubility parameter closer to that of diF-TESADT than PMMA, was used as a binder, the centro-apical phase separation was not observed in the printed line, as shown in Fig. 7a. This implies that the phase separation behavior in a printed blend solution was preferentially governed by the blend miscibility. Another factor in the vertical-phase separation to be considered is the surface energy of the blending components. If the polymer phase has a substrate preference, an initial phase separation can occur in a surface-oriented manner, forming a bilayer structure. PMMA commonly wets on the silicon substrate due to the strong interactions between the carbonyl groups of PMMA and the silanol groups on the silicon wafer^28^, Thus, the PMMA component of the blends preferentially migrates onto the silicon substrate. When the bottom-contact OFET devices consisting of source-drain electrodes located at the substrate surface were tested, most of the devices did not show electrical conductivity. (Fig. S9a in SI). This supports that the insulating PMMA component was preferentially deposited at the silicon surface. Additionally, in the phase-separation process, the component with the lower surface energy tends to segregate at the top surface to reduce the interfacial tension at the air/film interface. Considering that diF-TESADT has a larger water contact angle of 106° compared to that of PMMA (74°), one can reasonably state that the diF-TESADT moves toward the air/film interface during solvent evaporation. In the device fabrication process, the substrates with Au gate electrode were experimentally exposed to a UV-ozone atmosphere to make the substrate surface hydrophilic. When the blend solution was printed on the UV-ozone treated substrate, the cento-apical segregation of diF-TESADT with discrete phase separation boundary was observed on the Au electrode surface, where the diF-TESADT phase was completely removed after dipping in cyclohexane. (Fig. S9b in SI) Whereas, in the case of the untreated substrate, phase separation boundary was not clear and the diF-TESADT crystals remained after dipping in cyclohexane, indicating that the diF-TESADT domains were embedded in PMMA phase. These results imply that the surface energy compatibility between the blend components and the surface energy of the substrate is one of the important factors in the centro-apical phase separation. Furthermore, printing of the TIPS-PEN:PMMA blend was tested to investigate whether centro-apical phase-separation can occur in other small-molecule semiconductor/polymer blend system. Considering the solubility parameter (18 ~ 19 MPa^1/2^) and surface energy (i.e., water contact angle ~93°) of TIPS-PEN, the surface migration of TIPS-PEN can be reasonably expected in the TIPS-PEN:PMMA blends. As shown in Fig. 7b,c, OM images and the height profiles measured before and after the selective removal of TIPS-PEN clearly indicated that the TIPS-PEN molecules were also centro-apically segregated in the printed line. This result implies that the centro-apical movement of the small molecule semiconductor in a blend droplet is primarily affected by the vertical phase-separation condition.

**Figure 8:**
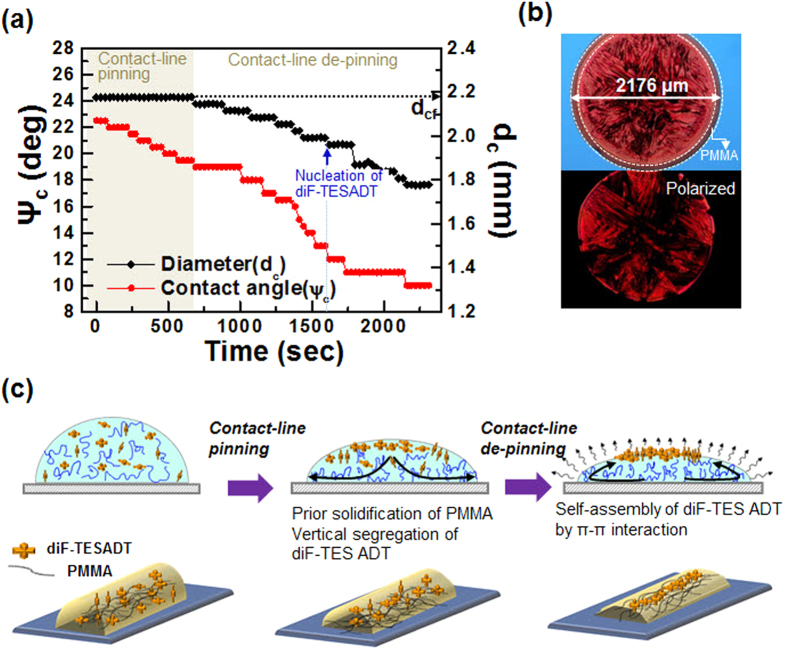
(**a**) Time-dependent variation of the contact angles(Ψ_c_) and the diameters(d_c_) of the evaporating blend solution droplet. (**b**) OM image (top) and POM image (bottom) of the blend solution droplet. (**c**) Schematic of how centro-apical phase-separation occurs during the drying process.

Different from the spin-casting process in which a strong centrifugal force is loaded onto the blend solution in rapid solvent evaporation conditions, phase-separation in a printed cylindrical droplet can be influenced by the hydrodynamic flows inside of the droplet during the solvent evaporation process. To investigate the drying behavior, a 1:4 diF-TESADT:PMMA blend (10 wt%) droplet was deposited onto the silicon wafer, and the solvent evaporation was monitored from a horizontal view and changes in the contact angle and diameter of the droplet were extracted from the images recorded *in situ* (Fig. 8a). As shown in Fig. 8b, the OM images of the blend deposited after complete drying verified that the diameter of the blend droplet after the complete evaporation of the solvent (d_cf_) was identical to the initial contact diameter of the droplet. This implies that as the evaporation progressed, the contact angle of the droplet decreased, whereas its diameter remained nearly constant. This behavior has been referred to as a contact line pinning in the drying droplet^29^,^30^. This contact line pinning is attributed to the non-uniform solvent evaporation rate. Faster evaporation at the perimeter of the droplet compared to evaporation at the center regime induced a hydrodynamic flow of the solvent from the center of the droplet to the contact line to replenish the amount of solvent lost by evaporation. Then, this outward hydrodynamic flow transported the suspended materials inside of the droplet to the drop’s perimeter. Interestingly, OM and polarized OM images of the blend droplet deposit, shown in Fig. 8b, verified that the PMMA phase dominantly existed near the perimeter of the droplet and that the diF-TESADT crystal structure was formed at the center regime. This indicates that the phase-separated PMMA phase solidified first near the perimeter of the droplet through rapid evaporation at the edge of the droplet during the contact line pinning stage. The hydrodynamic outward flow induced by the contact line pinning commonly produces a “coffee-ring” deposit. However, the highly concentrated diF-TESADT:PMMA blend solution used in this study did not exhibit a coffee-ring deposit due to its high viscosity. In fact, the printed blend line from the low concentration solution, as shown in Fig. 3a, supported this explanation in which the centro-apical phase separation of diF-TESADT occurred when the coffee-ring deposit occurred. This result enables us to better understand the existence of the PMMA phase near the perimeter regime of the printed blend line. Then, as the solvent evaporation proceeds, the evaporation mode switched to depinned contact, in which both the droplet diameter and contact angle decreased. It has been reported that the hydrodynamic flow inside of the drying droplet in the contact-lined depinned manner was directed to the centerline of the droplet^31^,^32^. During this stage, the nucleation of diF-TESADT crystals occurred approximately 27 min after the droplet deposition (Fig. 8a). This result implies that the inward hydrodynamic flow transported the suspended diF-TESADT molecules toward the center-region of the droplet, and then, the concentrated diF-TESADT molecules at the center region crystallized by strong intermolecular π-π interactions. Figure 8c is the schematic illustration of how centro-apical phase -separation occurs in the printed blend solution as the solvent evaporation proceeds.

## Conclusions

We demonstrated a centro-apical phase separation of small-molecule semiconductors in the printed cylindrical polymer blend droplet. Film characterization conducted by using cross-section-SEM, EDX, TOF-SIMS, and XPS confirmed that the diF-TESADT in the printed diF-TESADT:PMMA blends vertically segregated and simultaneously self-assembled to form a crystalline structure near the centerline of the printed line. The thicknesses and widths of the centro-apically separated diF-TESADT were successfully controlled by varying the diF-TESADT and PMMA ratio, concentration and printing velocity. XRD analysis confirmed that the centro-apically segregated diF-TESADT molecules were crystallized with an edge-on orientation, which is the desired orientation for the efficient charge transport in OFET devices. With regard to the possible mechanism of the centro-apical self-organization of small molecule semiconductors in the printed blend film, the vertical phase-separation combined with the hydrodynamic flows inside of the drying droplet likely resulted in the prior solidification of the PMMA phase near the perimeter of the droplet and the concentration of diF-TESADT near the centerline. As a proof-of-concept, a centro-apically phase-separated bilayer structure of diF-TESADT:PMMA blend was demonstrated via a facile method to form the semiconductor and dielectric layer for OFETs in one-step. The average field-effect mobilities and the on/off current ratios were 0.33 cm^2^V^−1^s^−1^ and ~10^5^, respectively, which closely correspond with previously reported values of the devices based on spin-casted diF-TESADT:PMMA blends. Furthermore, we have succeeded in the fabrication of flexible devices with a direct-print organic semiconductor channel that is well-defined within channel-lengths of as low as 10 μm. This study shows that the phase-separation behavior in the printed organic semiconductor/polymer blend is different from the spin-casted thin films and can be a key factor for the formulation of printable organic semiconductor ink.

## Methods

### Materials and Sample Preparation

2,8-Difluoro-5,11-bis(triethylsilylethynyl)anthradithiophene (diF-TESADT) was purchased from Lumtec in Taiwan and used without any further purification. Poly(methyl methacrylate) (PMMA M_w_ = 996 kgmol^−1^), and 1,2,4-trichlorobenzene were purchased from Sigma Aldrich Co. diF-TESADT and PMMA (1:1, 1:2, 1:4, 1:6, and 1:8 w/w ratio) were dissolved in 1,2,4-trichlorobenzene to produce 10 wt% solutions. A new batch of diF-TESADT and PMMA with a fixed 1:6 w/w ratio was dissolved in 1,2,4-trichlorobenzene to make 1, 3, 5, 8, and 10 wt% solutions. The diF-TESADT:PMMA blend solutions were printed into lines using a picoliter fluidic dispenser (SonoPlot Inc.) with a 50 μm orifice glass tip on a heavily doped n-type silicon wafer (with a 300 nm thick thermally grown SiO_2_ layer) under ambient room temperature conditions. The blend solutions were ejected and drawn from the glass tip by controlling the piezoelectrics. The line printings were performed by moving the glass tip across the wafer at speeds of approximately 70, 200, 400, and 1,000 μm/s. After printing, the samples were transferred to a vacuum oven at 60 °C and left overnight to remove the residual solvent. For the selective removal of the diF-TESADT layers, the printed lines were soaked in cyclohexane.

### Device fabrication

For the top-contact/bottom gate TFTs, Au gate electrodes (Au 100 nm) were patterned on a glass or polyethersulfone (PES) substrate by thermal evaporation using a shadow mask with 50 μm line patterns. The substrate with Au gate electrodes were exposed to a UV-ozone atmosphere (*λ* = 254 nm, 28 mW/cm^2^) for 15 min to make the substrate surface hydrophilic. The 10 wt% diF-TESADT:PMMA (1:4 w/w ratio) blend solution was printed onto the Au gate electrodes once by using the picoliter fluidic dispenser with the 50 μm orifice glass tip. The printed lines of the blend solution covered most of the electrodes. After printing, the sample was transferred to a vacuum oven at 60 °C and left overnight to remove the residual solvent. Au source-drain electrodes (Au 100 nm) were then patterned on top of the printed lines by thermal evaporation through a shadow mask. The shadow mask was aligned to fit a diF-TESADT crystalline pattern under an optical microscope and fixed to the substrate using a magnet attached at the backside of the substrate during deposition of Au. The channel length (L) and width (W) of the device were approximately 25 μm and 50 μm, respectively.

### Characterization

The morphologies of the printed lines were characterized by optical microscopy (OLYMPUS BX51). The crystal images of the diF-TESADT printed lines were obtained with polarized optical microscopy [(POM), OLYMPUS BX51]. The printed line thickness was determined using a surface profiler (AlphaStep AS-IQ). Cross-sectional scanning electron microscopy (SEM) images were obtained by a Helios NanoLab 660 (FEI) apparatus with a dual beam focused ion beam/scanning electron microscopy (FIB/SEM) system. Energy dispersive X-ray spectroscopy (EDS) analysis was performed with the Helios NanoLab 660 apparatus equipped with an EDS analytical system (Apollo 40, EDAX). To obtain the depth profile of the diF-TESADT molecules in the diF-TESADT:PMMA printed lines, TOF-SIMS was performed on the lines by using a TOF-SIMS 5 (ION-TOF Münster, Germany). Ion bombardment was used to slowly sputter material in an area of 200 × 200 μm^2^. The analysis was made on the 30 × 30 μm^2^ area of the printed lines. The contact angle was measured by a contact angle analyzer (8S150). The chemical analysis of the printed lines was performed by X-ray photoelectron spectroscopy (XPS) (PHI 5000 Versaprobe, ULVAC-PHI) with a monochromator Al Kα (1486.6 eV) X-ray source. Two-dimensional grazing-incidence X-ray diffraction (2D-GIXD) was measured at the Pohang Accelerator Laboratory in Korea (Beamline 9A, wavelength of x-ray source = 1.1159 Å). OFET characterization was conducted using an Agilent B1500A semiconductor device analyzer under ambient conditions. The capacitance (C_*i*_) of the printed PMMA lines sandwiched between the top and bottom electrodes was determined by using an Agilent 4284A precision LCR meter under ambient conditions.

## Additional Information

**How to cite this article**: Jin Lee, S. *et al.* Centro-Apical Self-Organization of Organic Semiconductors in a Line-Printed Organic Semiconductor: Polymer Blend for One-Step Printing Fabrication of Organic Field-Effect Transistors. *Sci. Rep.* **5**, 14010; doi: 10.1038/srep14010 (2015).

## Supplementary Material

Supplementary Information

Supplementary Video 1

## Figures and Tables

**Table 1:** Solubility parameter and water contact angle values of each component.

	Small molecule semiconductors		Insulating polymers	
	diF-TESADT	TIPS-PEN	PMMA	PS
Solubility parameter (MPa^1/2^)	15.5^a^	18 ~ 19^b^	22.6	17.5
Water Contact angle (deg)	106°	93°	74°	84°

^a^Solubility parameter for the diF-TESADT that was calculated by using the following equation and the group contribution method: *δ = *ρ*∑G/M* where *ρ* is the density, *M* is the molecular weight and *G* is the group molar attraction.(see Supporting Table S1).

^b^Reference by J. Chen, D. C. Martin, *J. Mater. Res.* **2007**, *22*, 1701.
