# Supplementary material for: Centro-Apical Self-Organization of Organic Semiconductors in a Line-Printed Organic Semiconductor: Polymer Blend for One-Step Printing Fabrication of Organic Field-Effect Transistors
Source: Sci Rep. 2015 Sep 11;5:14010. doi: 10.1038/srep14010 (PMC4566135; doi:10.1038/srep14010)
Supplement: Supplementary Information [file srep14010-s1.pdf]

Supplementary Information
for *Scientific Reports*

Centro-Apical Self-Organization of Organic Semiconductors in a Line-Printed Organic Semiconductor:Polymer Blend for One-Step Printing Fabrication of Organic Field-Effect Transistors

Su Jin Lee, Yong-Jae Kim, So Young Yeo, Eunji Lee, Ho Sun Lim, Min Kim, Yong-Won Song, Jinhan Cho, Jung Ah Lim*

Cross-sectional SEM analysis of the printed blend film

Figure S1(a) shows the top view (top) and cross-sectional (bottom) images of the printed pattern, and the portion marked by the red box ① covers half of the line-pattern width. The cross-sectional image shows a larger height at the center-line, where the diF-TESADT crystal was assumed to have formed, as previously noted. Energy dispersive spectroscopy (EDS) measurements were taken in the cross-sectional images of the regions marked by the red boxes ② and ③. The data taken from region ② are in **Figure S1(b)**, which is the side of the center crystalline part; there was no distinct phase separation, and sulfur that originated from diF-TESADT was not detected with the EDS. This indicates that the perimeter of the central diF-TESADT crystalline structure predominantly consisted of PMMA. **Figure S1(c)** shows the SEM image and EDX data taken from the region ③, which is the center crystalline part of the printed line. The cross-sectional image clearly shows the distinct layer boundary, which is derived from the vertical phase separation. EDS analysis focusing on each layer indicated that the top layer possessed a higher percentage weight of sulfur than that taken from the bottom layer. This result confirms that the diF-TESADT molecules were vertically concentrated at the air/film interface.

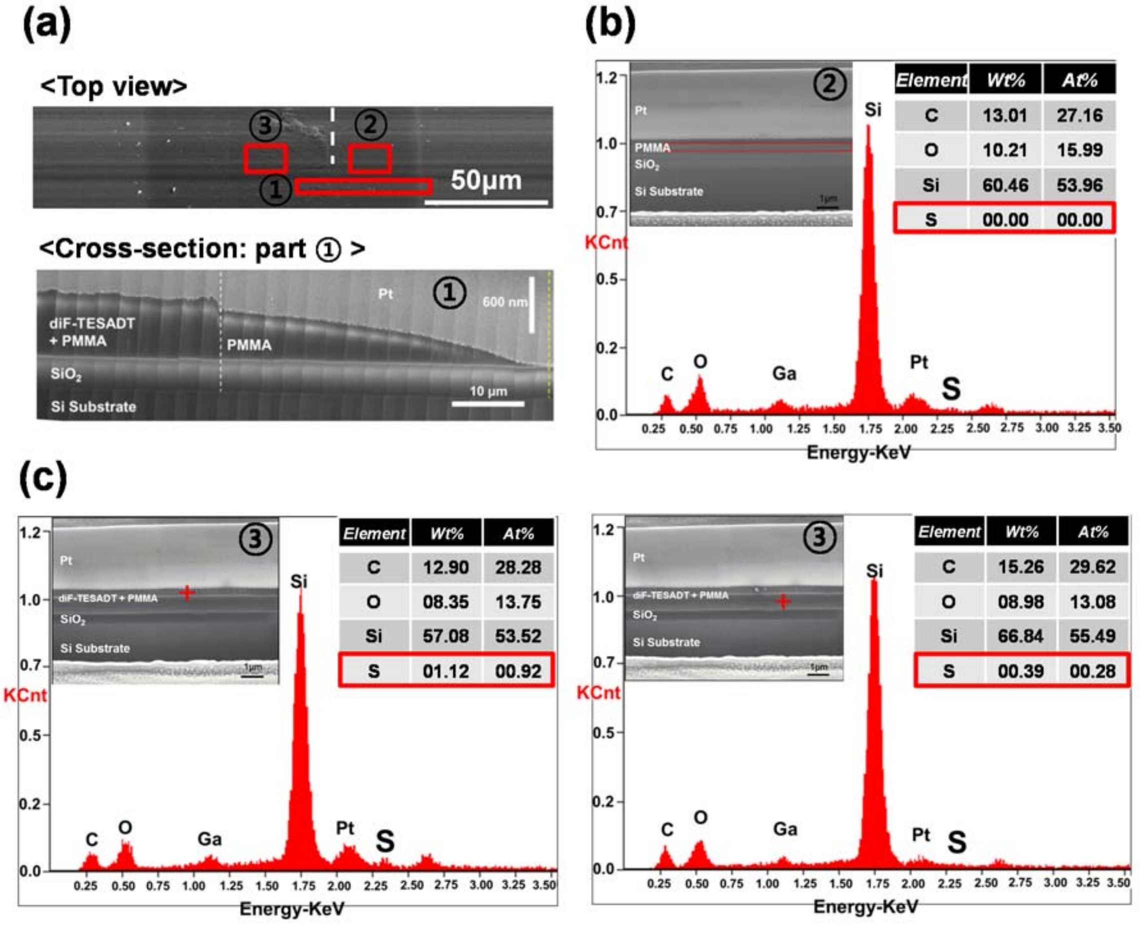

Figure S1. (a) Top view (top) SEM image of the entire printed line; ① is a magnified cross-section of the SEM image (bottom) marked by the red box ①. (b) EDS data of the red box ② region, which is the side of the center crystalline part and is assumed to be a PMMA layer. (c) EDS data of the red box ③ region, which is the center crystalline part of the printed line. Measurements were taken from the upper side and bottom side of the crystal line.

Time of flight secondary ion mass spectroscopy (TOF-SIMS) analysis

The silicon signals found in the chart were from the silicon wafer substrate. At the region close to the air/film surface (stage I), high intensities of the sulfur and fluorine signals from the diF-TESADT crystal were detected, whereas the oxygen signal from PMMA was extremely low. This result verified that the top layer of the central crystalline regime predominantly consisted of diF-TESADT molecules. Below the heavily segregated top layer, the sulfur and fluorine signals decreased, whereas the oxygen signal increased with a depth gradient. Subsequently, a PMMA-rich region with diF-TESADT as a minor component was observed near the substrate (stage II).

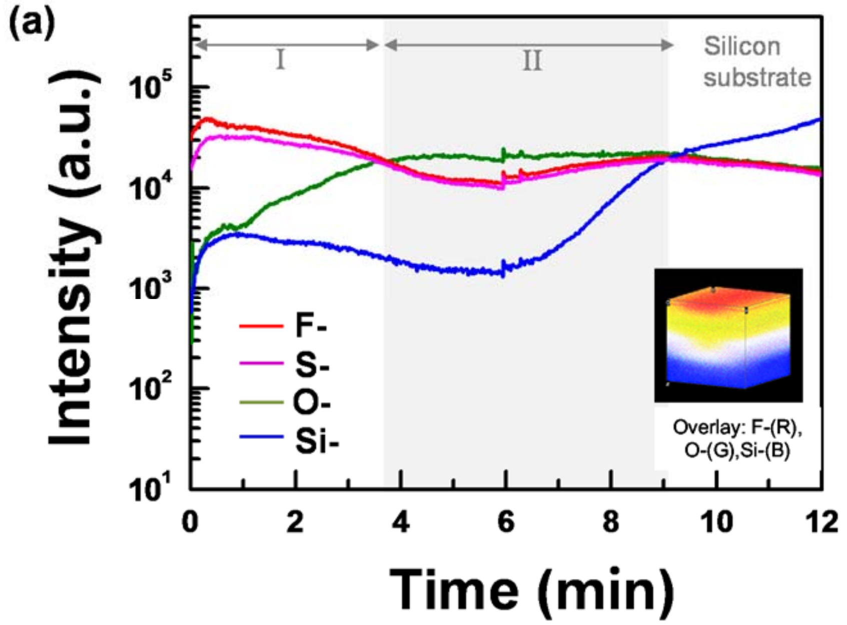

Figure S2. (a) Intensities of each element in the vertically segregated center region of the printed line as a function of sputtering time during TOF-SIMS. The analyzed area is 30 x 30μm^2. The inset image denotes the total of the intensity of each element with the 3D images.

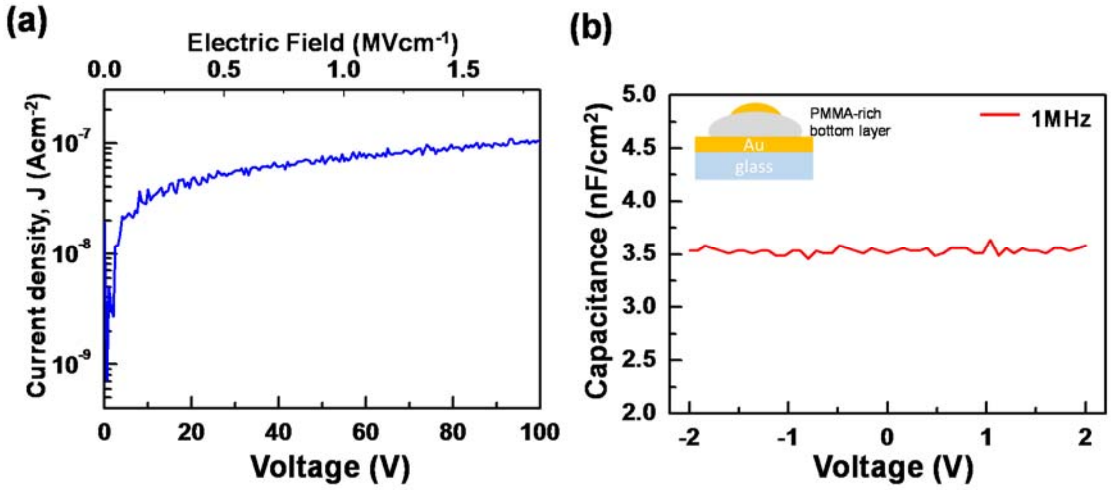

Figure S3 (a) Leakage current density versus voltage or electric field curve measurements for the PMMA-rich bottom layer, the diF-TESADT/PMMA printed line after dipping in cyclohexane. (b) Capacitance versus voltage for the PMMA-rich bottom layer, diF-TESADT/PMMA printed line after dipping in cyclohexane. The inset is a structure of the device used for the measurement.

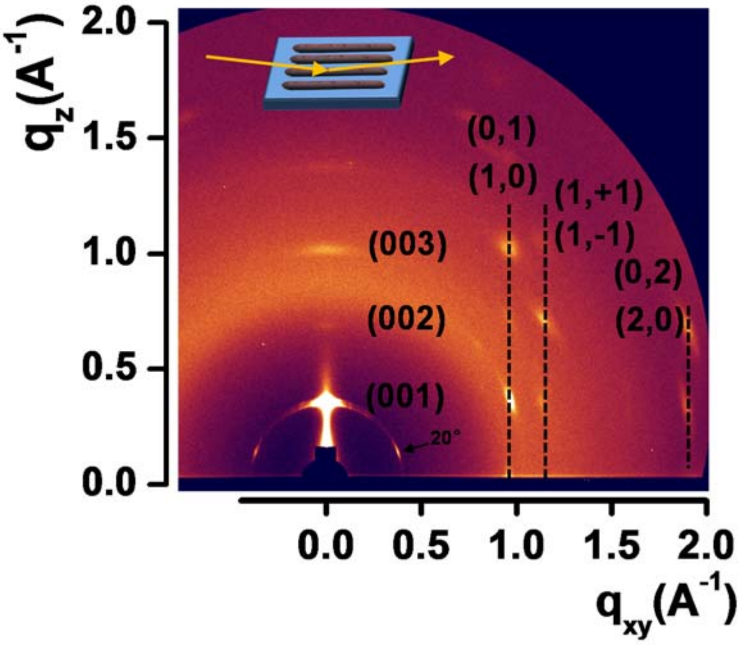

Figure S4. 2-dimensional grazing incidence X-ray diffraction (2D GIXD) patterns of the diF-TESADT/PMMA-blend printed line. The incident beam was injected parallel to the printed line.

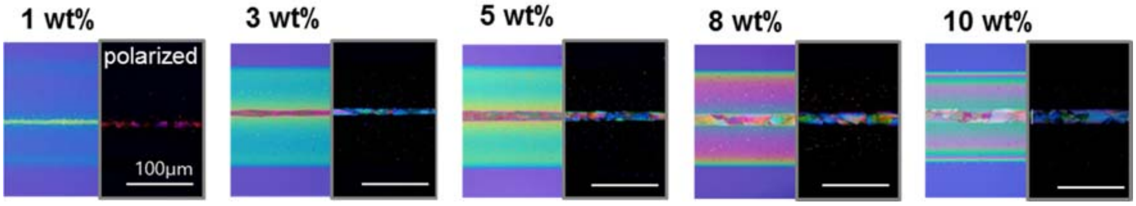

Figure S5. OM and POM images of line-printed 1:4 diF-TESADT:PMMA blend with a variation in the total solution concentration.

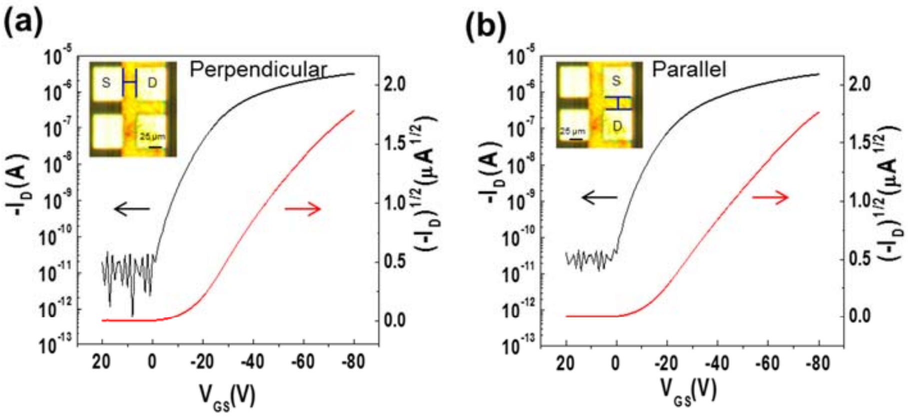

S/D arrangement	*Threshold voltage (V)*	*μ (cm²/Vs)*	*Ion/off*
Perpendicular	-12.0	0.20	~ 10^4
Parallel	-8.2	0.23	~ 10^4

*Figure S6.*Transfer (I_{DS}–V_{GS}) characteristics (at V_{DS} = −80 V) of a diF-TESADT:PMMA (1:4 w/w ratio) blend printed line transistors.with a source and drain arrangement (a) perpendicular and (b) parallel to the printing direction.

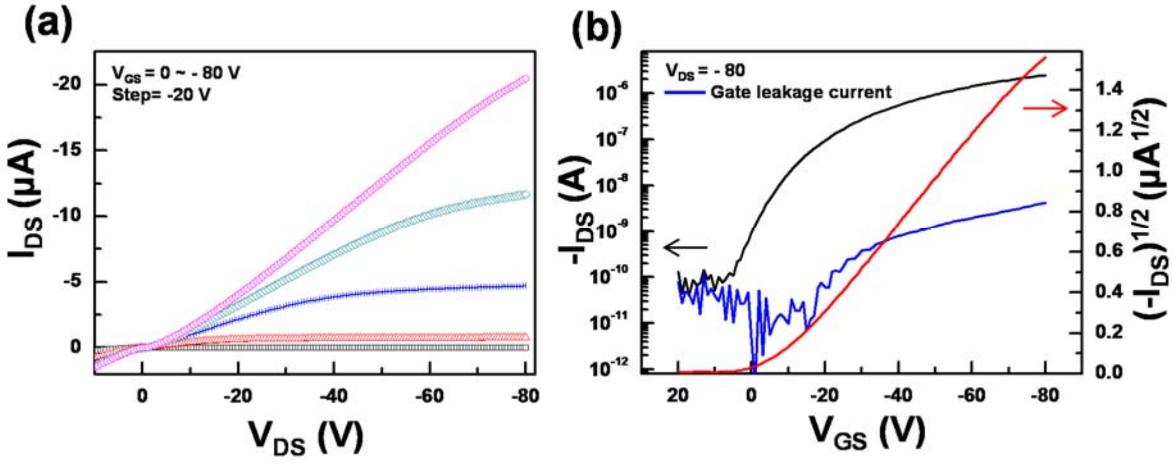

Figure S7 (a) The highest performance output ($I_{DS} - V_{DS}$) and (b) transfer ($I_{DS} - V_{GS}$) characteristics (at $V_{DS} = -80$ V) of a diF-TESADT:PMMA (1:4 w/w ratio) blend printed line transistor.

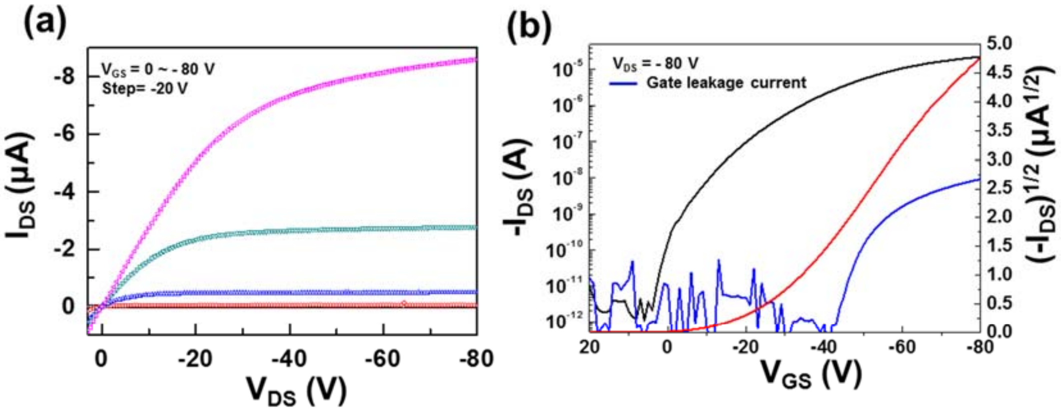

Sample	*Threshold voltage (V)*	*μ (cm2/Vs)*	*Ion/off*
Glass substrate	-23.1	0.054	~ 10^5
Flexible PES substrate	-12.6	0.057	~ 10^4

Figure S8 (a) The output ($I_{DS} - V_{DS}$) and (b) transfer ($I_{DS} - V_{GS}$) characteristics (at $V_{DS} = -80$ V) of a diF-TESADT:PMMA (1:8 w/w ratio) blend transistor fabricated on a glass substrate.

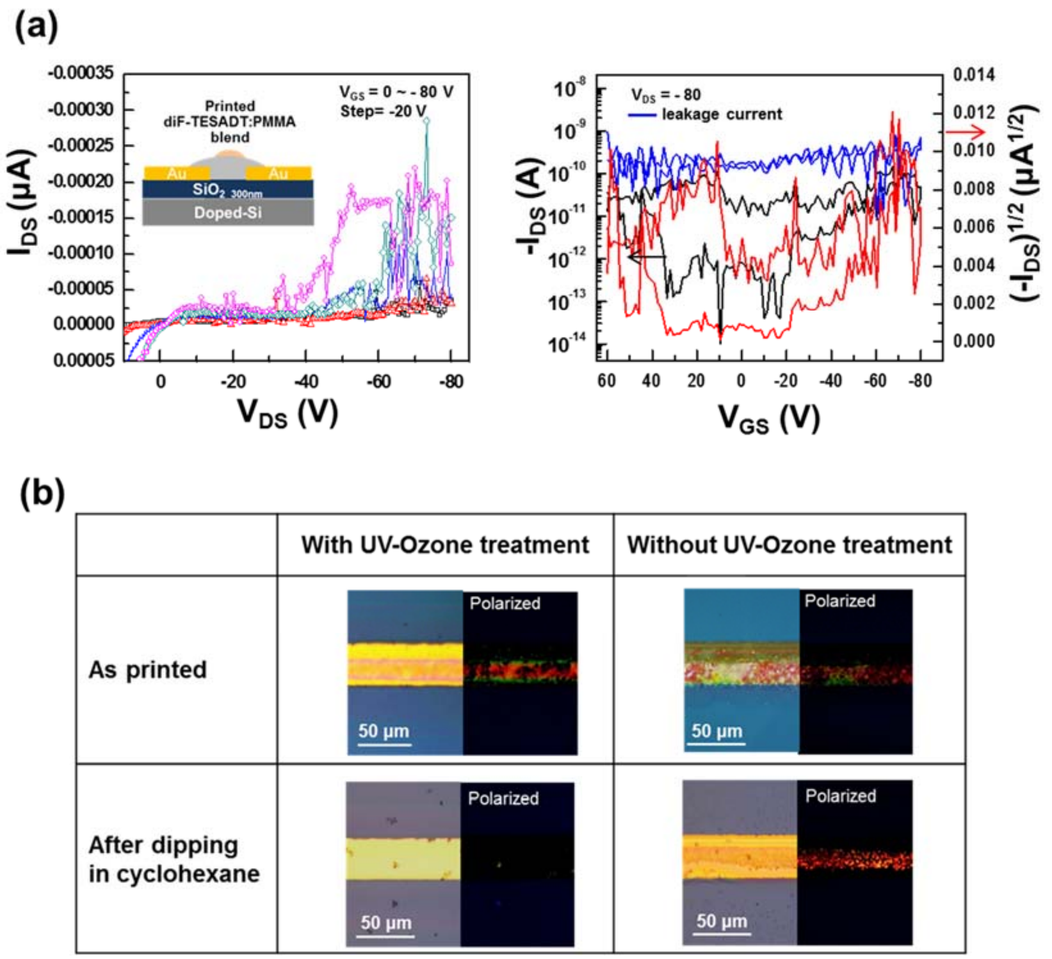

Figure S9. (a) The output ($I_{DS} - V_{DS}$) and transfer ($I_{DS} - V_{GS}$) characteristics (at $V_{DS} = -80$ V) of a diF-TESADT:PMMA (1:4 w/w ratio) blend transistor with bottom-contact source and drain electrodes. (b) Comparison OM and POM images of line-printed diF-TESADT:PMMA (1:4 w/w ratio) blend according to the UV-ozone treatment of the substrates..

Supporting table S1. Group contributions to cohesive energy density.[a)]

Group	F [$MPa^{1/2}$ cm^3/mol]
$-CH_3$	437
$>CH_2$	272
-Si	-77
-C≡C-	454
-F	250
-S	460
p-Phenylene	1350
-C=CH-	265

a) Group contribution values taken from reference [27]
